# Supplementary material for: Docosahexaenoic Acid and Arachidonic Acid Levels Are Associated with Early Systemic Inflammation in Extremely Preterm Infants
Source: Nutrients. 2020 Jul 5;12(7):1996. doi: 10.3390/nu12071996 (PMC7400618; doi:10.3390/nu12071996)
Supplement: Supplementary file 1 [file nutrients-12-01996-s001.pdf]

Supplement

A

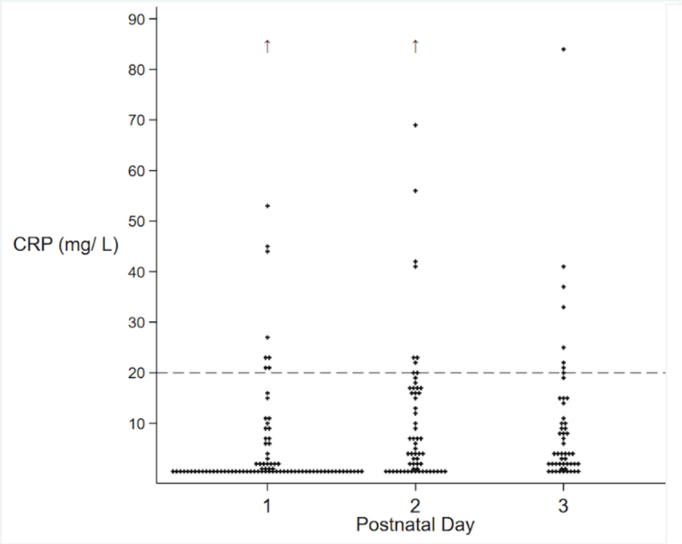

B

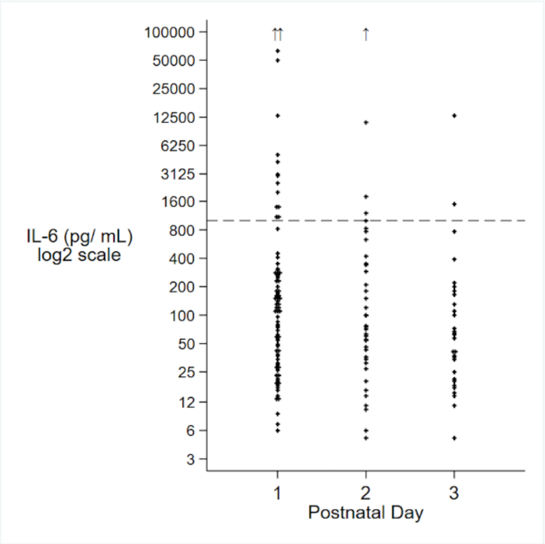

Figure S1. Distribution of clinical samples of (A) CRP, and (B) IL-6 the first three postnatal days.

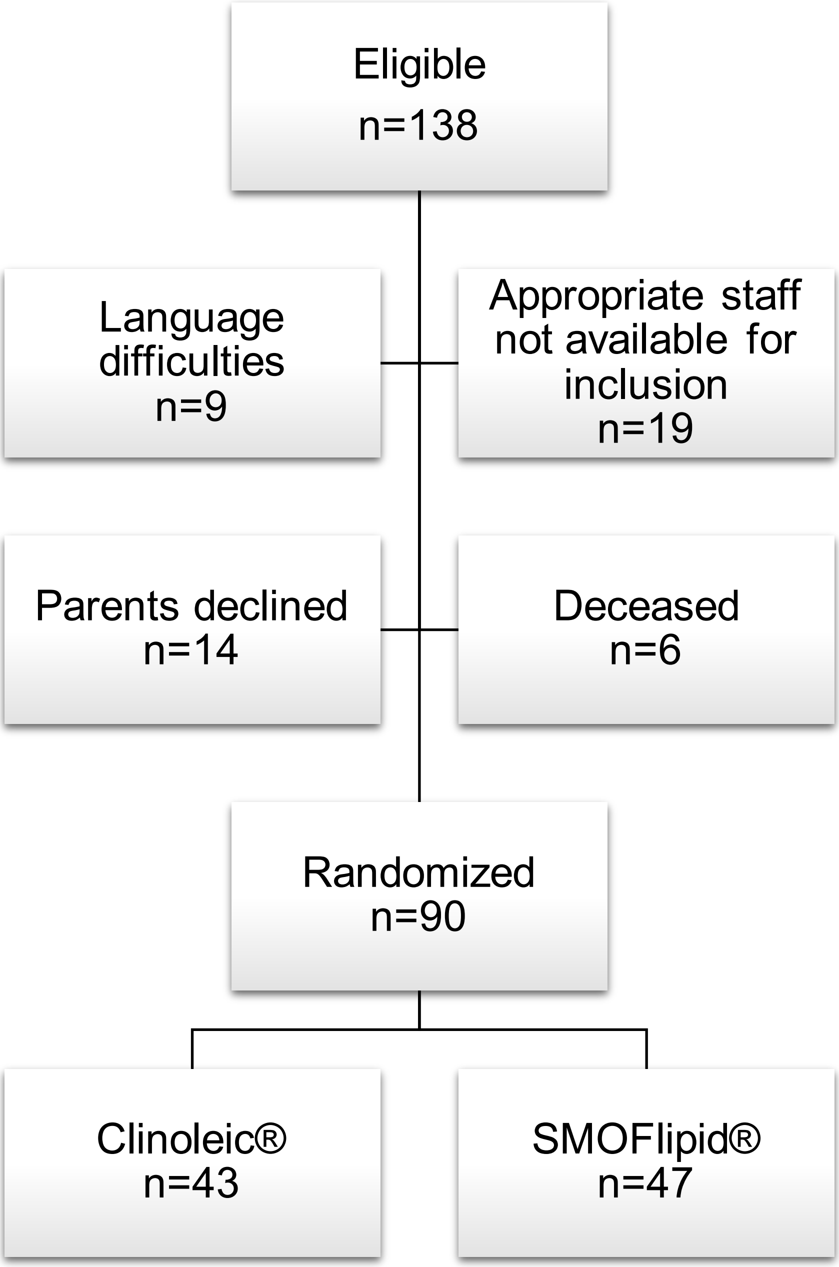

Figure S2. Patient enrollment flow chart.

Table S1. Fatty acid levels in cord blood, the first postnatal day, week and month.

	**Cord blood**<br>n=40	**Day 1**<br>n=90	**Day 7**<br>n=84	**Day 8-28***<br>n=72
omega-3<br>fatty acids				
ALA<br>mol%	0.02 (0.03)<br>0.01 (0.01-0.02)	0.10 (0.32)<br>0.04 (0.02-0.09)	0.12 (0.13)<br>0.10 (0.08-0.13)	0.10 (0.06)<br>0.09 (0.07-0.12)
EPA<br>mol%	0.52 (0.24)<br>0.46 (0.36-0.66)	0.60 (0.09)<br>0.55 (0.41-0.68)	0.91 (0.61)<br>0.83 (0.37-1.45)	0.91 (0.39)<br>0.84 (0.61-1.14)
DHA<br>mol%	3.30 (0.86)<br>3.30 (2.74-3.66)	3.23 (0.84)<br>3.21 (2.69-3.76)	2.22 (0.49)<br>2.16 (1.88-2.54)	2.49 (0.50)<br>2.50 (2.07-2.90)
omega-6<br>fatty acids				
LA<br>mol%	6.39 (1.35)<br>6.34 (5.56-6.92)	7.25 (2.13)<br>6.89 (5.90-8.10)	14.79 (2.58)<br>14.82 (13.51-16.59)	14.02 (2.04)<br>14.24 (12.47-15.73)
AA<br>mol%	16.17 (2.42)<br>16.39 (14.65-18.12)	14.58 (2.52)<br>14.53 (13.39-15.86)	8.00 (1.37)<br>7.95 (7.03-9.04)	7.94 (1.27)<br>7.94 (7.13-8.79)

Mean (SD), median (25th –75th percentile)

* Area under the curve, calculated by the trapezoidal rule using all samples and sampling days during the period of interest, divided by time to generate average fatty acid levels.
